# Evaluation of a microencapsulated form of zinc oxide on weanling pig growth performance, fecal zinc excretion, and small intestinal morphology

**DOI:** 10.1093/tas/txad146

**Published:** 2023-12-23

**Authors:** Payton L Dahmer, Cassandra K Jones, Franco M Ferreyra

**Affiliations:** Department of Animal Sciences and Industry, Kansas State University, Manhattan, KS, USA; Department of Animal Sciences and Industry, Kansas State University, Manhattan, KS, USA; Veterinary Diagnostic Lab, Kansas State University College of Veterinary Medicine, Manhattan, KS, USA

## Abstract

A total of 300 pigs (DNA 200 × 400; initially 6.0 ± 0.08 kg body weight [**BW**]) were used in a 42-d study to evaluate a microencapsulated form of zinc oxide. At weaning, pigs were randomly allocated to pens, and pens were randomly assigned to dietary treatments with 5 pigs per pen and 12 pens per treatment. Dietary treatments were 1) negative control (CON; standard nursery diet containing 110 ppm Zn in the form of zinc sulfate from trace mineral premix); 2) control diet with 400 ppm added Zn from ZnO included in phases 1 and 2 (Low-ZnO); 3) control diet with 3,000 ppm added Zn from ZnO included in phase 1 and 2,000 ppm added Zn from ZnO included in phase 2 (High-ZnO); 4) control diet with 400 ppm added Zn from microencapsulated ZnO included in phases 1 and 2 (Low-MZnO; Vetagro S.p.A., Reggio Emilia, Italy); 5) control diet with 3,000 ppm added Zn from microencapsulated ZnO in phase 1 and 2,000 ppm added Zn from microencapsulated ZnO in phase 2 (high-MZnO; Vetagro S.p.A., Reggio Emilia, Italy). On days 10 and 28, fecal samples from 2 pigs per pen were collected for fecal Zn concentrations, and on day 28, 30 pigs (*n* = 6) were euthanized, and small intestinal tissues were collected to evaluate morphology. For the entire treatment period (days 0 to 28) there was no evidence of differences in average daily gain (**ADG**), average daily feed intake (**ADFI**), or G:F (*P *> 0.05). During the common phase 3 (days 28 to 42) pigs fed the negative control, High-MZnO, or Low-MZnO had improved (*P *< 0.0001) ADG and ADFI compared to pigs fed High- or Low-ZnO. For the entire experiment (days 0 to 42), pigs fed Low-ZnO or High-ZnO had reduced (*P *< 0.0001) ADG compared to those fed the negative control. A significant treatment × day interaction (*P *= 0.04) was observed for fecal Zn concentrations, where the level of Zn excreted in the feces was dependent on the sampling day in pigs fed a low level of ZnO or low level of microencapsulated ZnO. There was no evidence (*P *> 0.05) that small intestinal morphology differed significantly between treatments. In summary, feeding a microencapsulated form of ZnO did not alter piglet growth performance during the treatment period. Pigs fed a low level of ZnO or microencapsulated ZnO had reduced fecal Zn excretion by the end of the feeding period, but no significant impacts were observed on piglet small intestinal morphology.

## Introduction

The postweaning period is one of the most crucial times in swine production as the stress induced on the young pig can negatively impact performance and health, sometimes resulting in mortality. During this time when the piglet’s digestive capabilities are still limited, the animal is drastically shifted from a liquid milk diet to solid feed, comingled with other pigs, and potentially exposed to pathogens (Campbell et al., 2013; Gebhardt et al., 2020; Wensley et al., 2021). Collectively, these nutritional and environmental changes can lead to a decrease in feed intake, subsequent performance, and clinical illness such as postweaning diarrhea (**PWD**). While various nutritional and management strategies have been implemented to help lessen the negative ramifications of the postweaning period, the addition of supplemental Zn in the form of ZnO to the diet has been proven one of the most effective (Sales, 2013). While the weanling piglet’s Zn requirement is around 100 mg/kg (NRC, 2012), when included at much higher levels, near 2,000 to 4,000 ppm, improvements in feed intake, growth performance, and fecal consistency have been determined (Hill et al., 2001; Shelton et al., 2011). These inclusion levels, termed pharmacological, are typically used for the first 2 wk postweaning. While the positive benefits of this feeding practice are well documented, there is scrutiny on the swine industry to reduce its reliance on pharmacological ZnO for various reasons. Since ZnO has a relatively low bioavailability, the excess Zn not absorbed by the pig is excreted in the manure that is eventually applied to the soil as fertilizer. Over time, there is potential for Zn to accumulate in the soil and pose a risk to the environment. In addition to potential environmental concerns, studies have demonstrated a possible linkage of pharmacological ZnO to the development of antimicrobial-resistant genes. Collectively, these issues have prompted regulatory action in some countries. For instance, the European Union placed a limit of 150 ppm total dietary Zn in 2022 (Commission, 2016). While these measures have not been enforced globally yet, the swine industry is still constantly evaluating other avenues to improve postweaning growth performance and PWD in the instance that pharmacological ZnO supplementation becomes obsolete.

One method to reduce ZnO inclusion that could potentially still provide the positive benefits associated with ZnO supplementation is the use of microencapsulated ZnO. As this technology further develops, the hypothesis that lower inclusion of microencapsulated ZnO can yield similar benefits to pharmacological levels of ZnO has been examined, but on few accounts. While there is some data to suggest the potential for microencapsulated ZnO to serve as a viable alternative to pharmacological ZnO (Grilli et al., 2015; Cho et al., 2018), there is still variability in the responses observed among literature, primarily due to differences in inclusion levels, methodologies for microencapsulation of the ZnO, and the age and existing health status of experimental animals. Thus, our objective was to further evaluate feeding a microencapsulated form of ZnO in place of pharmacological ZnO. Specifically, we aimed to determine the effects of microencapsulated ZnO on piglet growth performance, fecal zinc excretion, and small intestinal morphology.

## Materials and Methods

### Animals and Diets

All experimental procedures adhered to guidelines for the ethical and humane use of animals for research according to the Guide for the Care and Use of Agricultural Animals in Research and Teaching (FASS, 2012) and were approved by the Institutional Animal Care and Use Committee at Kansas State University (IACUC #4678). The experiment was conducted at the Kansas State University Swine Teaching and Research Center in Manhattan, Kansas.

A total of 300 pigs (DNA 400 × 200; Columbus, NE, initially, 6.0 ± 0.08 kg body weight [**BW**]) were weaned at an average of 21 d of age and used in a 42-d experiment. Weaning was considered day 0 of the trial and at this point, pigs were individually weighed and allotted to pens in a completely randomized design based on initial BW. Due to an uneven distribution of male and female piglets, we did not allocate pigs to pens in a way that genders were equally represented among treatments and pens. There were 5 pigs per pen and 12 replicate pens per treatment. Each pen had tri-bar floors (1.5 × 1.5 m) and was equipped with a 4-hole dry self-feeder and nipple waterer to supply ad libitum access to feed and water. At weaning, pigs were randomized to pens, and pens were randomly assigned to one of five dietary treatments. Diets were formulated and manufactured in three dietary phases (phase 1 = days 0 to 10; phase 2 = days 11 to 28; phase 3 = days 29 to 42) such that experimental diets were fed from days 0 to 28, and a common diet was fed to all pigs from days 28 to 42. Diets were fed in pelleted form in phase 1 and in mash form in phases 2 and 3. Diets were formulated to meet or exceed (NRC, 2012) requirements (Table 1). Treatments were as follows: 1) negative control (CON; standard nursery diet containing 110 ppm Zn in the form of zinc sulfate from trace mineral premix); 2) control diet with 400 ppm added zinc from ZnO included in phases 1 and 2 (Low-ZnO); 3) control diet with 3,000 ppm added zinc from ZnO included in phase 1 and 2,000 ppm added zinc from ZnO included in phase 2 (High-ZnO); 4) control diet with 400 ppm added zinc from microencapsulated ZnO included in phases 1 and 2 (Low-MZnO; Vetagro S.p.A., Reggio Emilia, Italy); 5) control diet with 3,000 ppm added zinc from microencapsulated ZnO in phase 1 and 2,000 ppm added zinc from microencapsulated ZnO in phase 2 (High-MZnO; Vetagro S.p.A., Reggio Emilia, Italy). A hydrogenated vegetable oil was used as the matrix for microencapsulation of the ZnO. All test ingredients were included at the expense of corn in the diet. Individual pig weights and feed disappearance were measured on days 0, 10, 14, 21, and 28, with pen weights collected on day 35, and 42 to calculate average daily gain (**ADG**), average daily feed intake (**ADFI**), and feed efficiency (**G:F**).

**Table 1.** Composition of phase 1, phase 2, and phase 3 basal diets (as-fed basis)

	Dietary phase^1^		
Item	Phase 1	Phase 2	Phase 3
Ground corn	44.61	49.71	65.65
Soybean meal, 47.5% CP	17.83	24.60	28.30
Whey permeate, 80% lactose	10.00	—	—
Spray dried whey	10.00	10.00	—
Corn DDGS, 7.5% oil	5.00	7.50	—
Enzymatically treated soybean meal^2^	4.00	3.50	—
Fish meal	2.50	—	—
Spray dried bovine plasma	2.00	—	—
Choice white grease	1.00	1.00	2.00
Calcium carbonate, 38.5% Ca	0.60	0.85	0.75
Monocalcium phosphate, 21.5% P	0.75	0.85	1.10
Salt	0.30	0.50	0.60
L-Lys HCL	0.45	0.55	0.55
DL-Met	0.20	0.22	0.21
L-Thr	0.19	0.20	0.23
L-Trp	0.03	0.04	0.05
L-Val	0.10	0.08	0.16
Vitamin premix w/ phytase^3^	0.25	0.25	0.25
Trace mineral premix^4^	0.15	0.15	0.15
Choline chloride, 60%	0.04	—	—
Zinc oxide	Varied	Varied	—
Zincoret S^5^	Varied	Varied	—
Total	100.00	100.00	100.00
Calculated analysis^6^			
SID amino acids, %			
Lys	1.40	1.35	1.30
Ile:Lys	56	56	53
Leu:Lys	114	119	111
Met:Lys	36	37	36
Met and Cys:Lys	57	58	57
Thr:Lys	65	63	63
Trp:Lys	18	19	19.3
Val:Lys	72	67	70
His:Lys	33	34	35
Total Lys, %	1.56	1.51	1.43
ME, kcal/kg	3,321	3,359	3,383
NE, kcal/kg	2,512	2,134	2,534
SID Lys:NE, g/Mcal	4.10	4.02	5.13
CP, %	21.7	21.2	19.9
Ca, %	0.71	0.70	0.65
P, %	0.65	0.63	0.61
STTD P, %	0.46	0.40	0.49

^1^Treatment diets were fed to 300 pigs (DNA 400 × 200 [Columbus, NE]; initially 6.03 ± 0.08 kg BW) for 28 d in a 2-phase feeding program with 5 pigs per pen and 12 pens per treatment. A common phase 3 diet was fed to all pigs from days 29 to 42.

^2^HP300 (Hamlet Protein, Findlay, OH).

^3^Premix provided per kg of premix: Ronozyme HiPhos (DSM Nutritional Products, Basel, Switzerland) provided 2,027 FTU/kg of feed and an estimated release of 0.12% STTD P4,409,249 IU vitamin A; 551,156 IU vitamin D3; 17,637 IU vitamin E; 1,764 mg vitamin K; 15.4 mg vitamin B12; 19,842 mg niacin; 11,023 mg pantothenic acid; and 3,307 mg riboflavin.

^4^Premix provided per kilogram of premix: 110 g Fe from iron sulfate; 110 g Zn from zinc sulfate; 26.4 g Mn from manganese oxide; 11 g Cu from copper sulfate; 198 mg I from calcium iodate; 198 mg Se from sodium selenite.

^5^Zincoret S (Vetagro S.p.A., Reggio Emilia, Italy).

^6^Based on digestible nutrient and net energy contest of feed ingredients according to the NRC (2012).

### Chemical Analysis

Complete diet samples were collected from 10 different feeders per dietary treatment on days 0 and 21 and composite subsamples were analyzed for nutrient composition (Table 2). Complete diet samples of the phase 3 common diet were collected from 10 different feeders on days 21 and 42 and composite subsamples were analyzed for nutrient composition (Table 3). Assays included DM (method 930.15; AOAC, 2007), crude protein (**CP**) as N × 6.25 using the combustion method (Nitrogen Determinator; model TruMac N, Leco Corporation, St. Joseph, MI; method 990.03; AOAC, 2007), fat (method 2003.05; AOAC, 2006), acid detergent fiber (**ADF**) (ANKOM Tech. Method 200), Ca (method 985.01; AOAC, 2006), P (method 985.01; AOAC, 2006), and Zn (method 985.01; AOAC, 2006).

**Table 2.** Analyzed composition of phase 1 and phase 2 experimental diets (as-fed basis)^1^

	Dietary treatment^2^				
Analyzed composition, %^3^	CON	Low-ZnO	High-ZnO	Low-MZnO	High-MZnO
Phase 1					
DM, %	88.74	88.11	87.99	87.84	87.65
CP, %	19.00	18.30	18.9	18.90	19.5
Fat, %	3.53	3.53	3.43	3.43	3.83
ADF, %	2.60	2.50	2.40	2.50	2.60
Calcium, %	0.75	0.69	0.70	0.77	0.89
Phosphorous, %	0.64	0.62	0.63	0.69	0.71
Zinc, ppm	178	572	2210	458	2110
Phase 2					
DM, %	87.86	87.43	87.74	87.76	88.03
CP, %	21.5	20.8	21.5	21.5	19.4
Fat, %	3.33	3.26	3.61	3.47	3.85
ADF, %	4.60	4.90	4.40	4.40	4.30
Calcium, %	0.76	0.66	0.79	0.91	1.15
Phosphorous, %	0.55	0.51	0.60	0.62	0.59
Zinc, ppm	127	391	2060	782	1370

^1^Treatment diets were fed to 300 pigs (DNA 400 × 200 [Columbus, NE]; initially 6.0 ± 0.08 kg BW) for 28 d in a 2-phase feeding program with 5 pigs per pen and 12 pens per treatment.

^2^Dietary treatments included: negative control basal diet with no added ZnO (CON); basal diet with 400 ppm added Zn from ZnO in phases 1 and 2 (Low-ZnO); basal diet with 3,000 ppm added Zn from ZnO in phase 1 and 2,000 ppm added Zn from ZnO in phase 2 (High-ZnO); basal diet with 400 ppm added Zn from microencapsulated ZnO in phases 1 and 2 (Low-MZnO); and basal diet with 3,000 ppm added Zn from microencapsulated ZnO in phases 1 and 2,000 ppm added Zn from microencapsulated ZnO in phase 2 (High-MZnO).

^3^Complete diet samples were collected from the same 10 randomly selected feeders on days 0 and 21. Samples were pooled by day and subsampled, then submitted to Midwest Laboratories (Omaha, NE) for proximate and zinc analysis.

**Table 3.** Analyzed composition of phase 3 common diet (as-fed basis)^1^

Analyzed composition, %^2^	Diet
DM, %	86.07
CP, %	20.10
Fat, %	3.78
ADF, %	3.50
Calcium, %	0.73
Phosphorous, %	0.61
Zinc, ppm	103

^1^A common diet was fed to 300 pigs (DNA 400 × 200 [Columbus, NE]; initially 6.0 ± 0.08 kg BW) from days 29 to 42 with 5 pigs per pen and 12 pens per treatment.

^2^Complete diet samples were collected from the same 10 randomly selected feeders on days 28 and 42. Samples were pooled and subsampled, then submitted to Midwest Laboratories (Omaha, NE) for proximate and zinc analysis.

### Fecal Zinc Content

Fresh fecal samples were collected from the same 2 randomly selected pigs per pen on days 10 and 28 (end of dietary phases 1 and 2, respectively) via rectal massage for analysis of Zn concentrations. Samples were pooled on a per-pen basis to form one composite sample for each collection point and frozen at −20°C until sample preparation. Samples were dried in a 55°C forced-air oven for 48-h, ground to pass through a 1-mm screen, and shipped to a commercial laboratory (Midwest Labs, Omaha, NE) for zinc analysis (method 985.01).

### Small Intestinal Morphology

At the conclusion of dietary phase 2 (day 28 of the study), a subset of pigs (*n *= 6, 30 pigs total) closest to the treatment average BW, regardless of the pen, were euthanized via captive bolt for collection of small intestinal tissue samples for morphological analysis. Briefly, once opened via an abdominal incision, a 2 cm segment of ileum was harvested from the proximal portion of the ileum and another 2 cm segment was collected ~10 cm proximal from the ileocecal valve and fixed in 10% buffered formalin. Samples were stored at room temperature until further processing. A 4 µm cross-section of ileum was then trimmed, embedded, and stained with hematoxylin and eosin per animal, and ten consecutive full-sized villi located within the mesenteric border were identified for the analysis. When incomplete, folded, and/or damaged villi were observed within the area of analysis, they were excluded and a matching number of full-sized villi adjacent to the area of evaluation were included. For each villus, the villus height (**VH**) and crypt depth (**CD**) were measured using an Olympus BX53 bright light microscope coupled with a mounted Olympus DP47 camera and analyzed using Olympus cellSens 1.18 software (Olympus Life Sciences, Tokyo, Japan). The villus height-to-crypt depth ratio (VH:CD) was calculated. Assessment and measurement of villi were performed by a single veterinary pathologist.

### Statistical Analysis

All data were analyzed using the GLIMMIX procedure of SAS version 9.4 (SAS Institute, Cary, NC). Growth and fecal zinc data were analyzed as a completely randomized design with a pen of pigs as the experimental unit, while the individual pig was the experimental unit for intestinal morphology data. Growth and intestinal morphology data included treatment as a fixed effect in the model, while fecal Zn data included the main effects of dietary treatment, sampling day, and their interaction. All comparisons included Tukey–Kramer multiple comparison adjustments. Data were considered significant if *P *< 0.05 and a trend if 0.05 < *P *< 0.10.

## Results and Discussion

### Growth Performance

Growth performance data are presented in Table 4. No evidence of differences in any growth performance parameters was observed for the entirety of the treatment period (days 0 to 28). From days 29 to 35, all pigs were fed a common phase 3 diet that contained no supplemental ZnO. During this timeframe, we observed that pigs who had previously been fed conventional ZnO had poorer ADG (*P* < 0.0001) compared to pigs who were previously fed the negative control or either dose of microencapsulated ZnO. Pigs fed the negative control diet or microencapsulated ZnO had greater ADFI (*P* < 0.0001) relative to their contemporaries fed conventional ZnO. For the entirety of the experiment (days 0 to 42), pigs fed conventional ZnO at either a low or pharmacological dose, had reduced ADG (*P *< 0.0001) compared to pigs fed a negative control diet or a diet containing a low or high dose of microencapsulated ZnO. Pigs fed a high dose of microencapsulated ZnO had increased ADFI (*P *= 0.02) compared to those fed either level of conventional ZnO, while pigs fed the negative control or a low dose of microencapsulated ZnO were intermediate. Piglet BW was not significantly different across dietary treatments at experiment days 0, 10, or day 28; however, by day 35, pigs fed a high level of microencapsulated ZnO were heavier (*P *< 0.01) than those fed a high dose of conventional ZnO, with all other treatments being intermediate. By the end of the 42-d experiment, pigs fed either dose of conventional ZnO were lighter (*P *< 0.05) than pigs fed the negative control, or both levels of microencapsulated ZnO.

**Table 4.** Growth performance of pigs fed a diet containing a low or high level of added zinc in the form of either free zinc oxide (ZnO) or a microencapsulated zinc oxide^1^

	Dietary treatment^2^						
Item	CON	Low-ZnO	High-ZnO	Low-MZnO	High-MZnO	SEM	*P* value
BW, kg							
Day 0	6.00	6.06	6.00	6.07	6.07	0.083	0.94
Day 10	7.28	7.35	7.29	7.35	7.49	0.117	0.71
Day 28	14.22	13.90	13.92	14.60	14.62	0.296	0.24
Day 35	18.31^abc^	17.29^bc^	16.84^c^	18.57^ab^	18.89^a^	0.383	<0.01
Day 42	23.59^a^	21.40^b^	20.77^b^	23.65^a^	24.38^a^	0.477	<0.0001
Phase 1 (days 0 to 10)							
ADG, kg/d	0.13	0.13	0.13	0.13	0.14	0.008	0.63
ADFI, kg/d	0.18	0.21	0.21	0.22	0.21	0.010	0.13
G:F	0.68	0.61	0.62	0.60	0.70	0.033	0.14
Phase 2 (days 11 to 28)							
ADG, kg/d	0.36	0.32	0.36	0.38	0.37	0.010	0.81
ADFI, kg/d	0.55	0.58	0.52	0.53	0.51	0.025	0.27
G:F	0.66	0.64	0.71	0.72	0.74	0.039	0.28
Overall treatment (days 0 to 28)							
ADG, kg/d	0.30	0.28	0.28	0.30	0.30	0.009	0.26
ADFI, kg/d	0.45	0.46	0.44	0.45	0.45	0.013	0.70
G:F	0.66	0.62	0.65	0.67	0.68	0.017	0.12
Overall common (days 28 to 35)							
ADG, kg/d	0.67^a^	0.54^b^	0.51^b^	0.65^a^	0.69^a^	0.017	<0.0001
ADFI, kg/d	0.91^ab^	0.74^c^	0.77^bc^	0.95^a^	1.00^a^	0.036	<0.0001
G:F	0.74	0.73	0.67	0.69	0.69	0.024	0.19
Overall Experiment (days 0 to 42)							
ADG, kg/d	0.41^a^	0.36^b^	0.36^b^	0.41^a^	0.42^a^	0.010	<0.0001
ADFI, kg/d	0.60^ab^	0.55^bc^	0.54^c^	0.60^ab^	0.61^a^	0.018	0.02
G:F	0.70	0.67	0.65	0.68	0.68	0.015	0.38

^abc^Means within a row that do not share a common superscript differ significantly (*P *< 0.05).

^1^A total of 300 weanling pigs (DNA 400 × 200 [Columbus, NE]; initially 6.0 ± 0.08 kg BW) were used in a 42-d growth study with 5 pigs/pen and 12 replicates/treatment.

^2^Dietary treatments included: negative control basal diet with no added ZnO (CON); basal diet with 400 ppm added Zn from ZnO in phases 1 and 2 (Low-ZnO); basal diet with 3,000 ppm added Zn from ZnO in phase 1 and 2,000 ppm added Zn from ZnO in phase 2 (High-ZnO); basal diet with 400 ppm added Zn from microencapsulated ZnO in phases 1 and 2 (Low-MZnO); and basal diet with 3,000 ppm added Zn from microencapsulated ZnO in phases 1 and 2,000 ppm added Zn from microencapsulated ZnO in phase 2 (High-MZnO).

Typically, pharmacological supplementation of ZnO to the weanling pig diet takes place during the first few weeks postweaning (Canibe et al., 2022). While not perfectly understood, ZnO is thought to positively impact the pigs gastrointestinal tract and immune system during the postweaning period by increasing nutrient digestibility and enzyme secretion, enhancing small intestinal barrier integrity and absorptive capacity, regulating pro- and anti-inflammatory cytokines, and providing a slight antimicrobial effect (Bonetti et al., 2021). These proposed benefits have been cited as responsible for the benefits in growth performance with pharmacological ZnO supplementation immediately postweaning across the body of literature (Hill et al., 2001; Sales, 2013). In the current study, no enhancements in growth performance were seen during the first 28-d postweaning when pigs were fed pharmacological levels of conventional ZnO. While this type of response is quite uncommon among published literature, there are reports of either minimal or negative responses to pharmacological ZnO supplementation. For instance, in a study by Hill et al. (2001), early-weaned pigs (15 d of age) were fed 2,000 ppm ZnO and no growth response was observed during the first week postweaning. While weaning age has been shown to play a role in the piglet’s ability to overcome the weaning transition, pigs in the current study were weaned older than pigs in the study conducted by Hill et al. (2001). It is important to note that the analyzed values of Zn in both the High-ZnO and High-MZnO treatments were lower than formulated in phase 1 (3,000 ppm formulated vs. ~2,000 ppm analyzed), which could perhaps explain the lack of response seen immediately postweaning. There were also no discernable differences in growth performance as a result of feeding either level of a microencapsulated ZnO during this time. This refutes previous work by Grilli et al. (2015), where weanling pigs were fed 3,000 ppm ZnO and either 300 or 800 ppm microencapsulated ZnO. These authors reported similar growth performance in pigs fed microencapsulated ZnO to those fed pharmacological ZnO. However, similar to our findings, Shen et al (2014) observed no statistically significant differences in growth performance between pigs fed a microencapsulated form of ZnO or ZnO.

During the common period, pigs that had previously been fed a diet with conventional ZnO at either low or pharmacological levels had reduced growth performance compared to pigs previously fed the negative control or a diet with microencapsulated ZnO at low or high levels. There are extremely limited published data to corroborate the negative response in gain and feed intake of pigs previously fed conventional ZnO during the common phase. Studies by Batson et al. (2021) and Feldpausch et al. (2018) found that pigs fed conventional ZnO during an experimental period had poorer feed conversion after pigs were switched to a common diet, but no significant ramifications were observed on ADG or ADFI. In an experiment by Poulsen (1989), pigs were fed 4,000 ppm ZnO and had reductions in both ADFI and ADG, which were attributed to potential zinc toxicity. According to Burrough et al. (2019), pigs experiencing zinc toxicosis can have reduced ADG and G:F; however, chemical analysis of diets in the current experiment validate that Zn levels were substantially lower than that of Poulsen (1989), nor did pigs experience other clinical signs indicative of Zn overload, such as lameness, depression, or elevated serum zinc (data not shown). It is well understood that dietary supplementation with ZnO, especially at pharmacological levels, stimulates the secretion of ghrelin (Yin et al., 2009), which could explain why pigs experienced lower ADFI ZnO, regardless of form, was removed from the diet. However, we are unable to precisely explain this response. Further investigation into how a microencapsulated form of ZnO can impact weaned pig performance should be conducted.

### Fecal Zinc Content

Fecal Zn levels are shown in Table 5 and are presented as the ppm of Zn detected in the fecal sample on a DM basis. A significant sampling day × treatment interaction (*P *= 0.04) was observed for fecal Zn content, where the level of Zn excreted in the feces was dependent on the sampling day in pigs fed a low level of ZnO or low level of microencapsulated ZnO, while excretion of Zn did not differ between days 10 and 28 in pigs fed a negative control, high level of ZnO, or high level of microencapsulated ZnO.

**Table 5.** Effect of feeding microencapsulated zinc oxide (ZnO) on weanling pig fecal Zn excretion (ppm Zn, DM basis)^1^

	Dietary treatment^2^						
Sampling day	CON	Low-ZnO	High-ZnO	Low-MZnO	High-MZnO	SEM	Treatment × day, *P = *
Day 10	2,260^cde^	4,560^bc^	9,960^a^	5,830^b^	6,960^b^	629.5	0.04
Day 28	624^e^	1,791^de^	9,413^a^	2,659^cd^	6,045^b^		

^abc^Means within a row or within a column that do not share a common superscript differ significantly, *P *< 0.05.

^1^A total of 300 pigs (DNA 200 × 400 [Columbus, NE]; initially 6.03 ± 0.08 kg BW) were used in a 42-d experiment with 5 pigs per pen and 12 pens per treatment. On days 10 and 28, a fresh fecal sample was collected from the same two randomly selected pigs per pen for zinc analysis.

^2^Dietary treatments were: negative control basal diet with no added ZnO (CON); basal diet with 400 ppm added Zn from ZnO in phases 1 and 2 (Low-ZnO); basal diet with 3,000 ppm added Zn from ZnO in phases 1 and 2,000 ppm added Zn from ZnO in phase 2 (High-ZnO); basal diet with 400 ppm added Zn from microencapsulated ZnO in phases 1 and 2 (Low-MZnO); and basal diet with 3,000 ppm added Zn from microencapsulated ZnO in phases 1 and 2,000 ppm added Zn from microencapsulated ZnO in phase 2 (High-MZnO).

There are concerns that pharmacological ZnO supplementation can result in the accumulation of Zn in the soil when swine manure is applied as fertilizer. It has been shown that the bioavailability of the Zn from ZnO is much lower than Zn itself, thus, the portion of Zn not absorbed by the pig is then excreted in the feces (Poulsen and Larsen, 1995; Buff et al., 2005). Regulatory action has been taken in some parts of the world to limit ZnO supplementation at levels in excess of nutritional requirements such that the excretion of Zn in swine manure is reduced. One of the hypothesized advantages of microencapsulated ZnO is that lower inclusion levels can be fed compared to ZnO. In this case, inclusion of microencapsulated ZnO can be lower than the 2,000 to 4,000 ppm that are typically used when adding conventional ZnO to the weanling pig diet (Hill et al., 2001). Our study design inherently allowed for the direct comparison of ZnO and microencapsulated ZnO at two inclusion levels, either a low dose (400 ppm added Zn) or a pharmacological dose (2,000 and 3,000 ppm added Zn). We are unable to clearly explain the significant interaction observed in the current study. It is important to note the limitations of our methodology, as we did not account for Zn intake or differences in fecal output between treatments and across sampling days. Samples were analyzed on a DM basis and not adjusted for total fecal output. While we know that fecal excretion of Zn increases linearly with Zn intake (Hansen et al., 2023), future work evaluating Zn excretion should consider factors such as Zn intake and differences in fecal output.

### Small Intestinal Morphology

The VH, CD, and VH:CD ratios were evaluated in both the proximal and distal ileum. No evidence of differences in in proximal ileal VH, CD, and VH:CD were observed between dietary treatments. Likewise, there was no evidence of differences in distal ileal VH, CD, or VH:CD between dietary treatments. These data are not shown.

Studies have reported morphological improvements in the small intestine of weaned pigs fed pharmacological levels of ZnO, whereas the VH was increased (Li et al., 2001; Zhu et al., 2017). However, it is well-known that ZnO supplementation at pharmacological levels does not consistently increase the absorption of Zn (Poulsen and Larsen, 1995; Krebs, 2000). Therefore, it is theorized that microencapsulated ZnO can allow for a slower and more controlled release of Zn along the small intestine, thereby increasing its ability to be absorbed and impact intestinal health. Previous studies have tested this hypothesis and reported significant improvements in small intestinal morphology in pigs fed various doses of microencapsulated ZnO compared to negative control, with no differences relative to conventional ZnO fed at pharmacological doses (Grilli et al., 2015; Kim et al., 2015; Lei and Kim, 2018). Conversely, we observed no histological alterations in the ileum as a result of feeding both conventional ZnO and microencapsulated ZnO. The variability in results across the literature can be explained by differences in dosage, piglet health status, and experimental conditions. Pigs in the current work were housed in a research facility with no known health challenges, which could potentially explain why no differences in small intestinal morphology were observed.

## Conclusions

In summary, feeding ZnO or a microencapsulated form of ZnO at both low and high inclusion levels did not alter the growth performance of pigs immediately postweaning. However, pigs fed ZnO at both a low or high level had reduced performance during the late nursery compared to those fed either level of microencapsulated ZnO or a control diet. Additionally, small intestinal morphology was not impacted by supplementing either form of ZnO at both a low and high level. Fecal excretion of Zn across dietary treatments was dependent on the sampling day, but pigs fed pharmacological levels of ZnO had numerically greater excretion of Zn compared to all other treatments.
